# Biogenic selenium nanoparticles with antifungal activity against the wood-rotting fungus *Oligoporus pelliculosus*

**DOI:** 10.1016/j.btre.2023.e00787

**Published:** 2023-02-07

**Authors:** Micaela Pescuma, Francisca Aparicio, Roberto D. Zysler, Enio Lima, Claudia Zapata, Jorge A. Marfetán, M.Laura Vélez, Omar F. Ordoñez

**Affiliations:** a Centro de Investigación y Extensión Forestal Andino Patagónico (CIEFAP), Esquel, Chubut, Argentina; b CONICET Consejo Nacional de Investigaciones Científicas y Técnicas; c Universidad Nacional de la Patagonia San Juan Bosco (UNPSJB), Esquel, Chubut, Argentina; d Instituto de Nanociencia y Nanotecnología, CNEA-CONICET, San Carlos de Bariloche, Río Negro, Argentina

**Keywords:** Selenium nanoparticles, Selenite reduction, Wood rotting fungi, Green technology, Bioactive nanoparticles, Antifungal activity

## Abstract

Selenium nanoparticles (SeNPs) have antimicrobial and antifungal activity. SeNPs using Se resistant bacteria is a low cost and eco-friendly technology. Fungal contamination of wood during drying is one of the main causes of economic losses in the wood industry. The bacterium *Delftia* sp. 5 resistance to Se and its ability to produce SeNPs able to inhibit the growth of the wood brown-rotting fungus *Oligoporus pelliculosus* was analyzed*.* The strain showed an optimal SeNPs production when selenite concentration was 160 mg *L*^−1^. The SeNPs were spherical with an average size 192.33 ± 8.6 nm and a zeta potential of -41.4 ± 1.3 nm. The SeNPs produced by *Delftia* sp. 5 (33.6 ± 0.1 mg *L*^−1^ Se) inhibited the growth of *O. pelliculosus* in agar plates and in *Nothofagus pumilio* (Lenga) wood samples. *Delftia* sp. 5 SeNPs could be used for embedding lenga wood prior to drying for preventing the growth of the deteriorating fungi *O. pelliculosus*.

## Highlights

• *Delftia* sp. 5 produced selenium nanoparticles with antifungal activity.
• Selenium nanoparticles inhibit the growth of the wood-decay fungus *O. pelliculosus*.
• *Delftia* sp. 5 produced organic coated selenium nanoparticles.

## 1 Introduction

Among the various methods developed to obtain nanoparticles (NPs) with special sizes and shapes, and with unique characteristics when dispersed in different solvents, the biogenic routes are gaining much interest. For instance, metallic and non-metallic NPs can be produced using plant extracts and microorganisms. These approaches constitute a low-cost green technology [1], [2], [3], [4], [5], [6], [7]. In addition, biogenic NPs produced by bacteria have some advantages: they are known to be more stable and to have an enhanced antimicrobial activity over their synthetic counterparts, due to the presence of bacterial cell membrane components.

Biogenic SeNPs production can be achieved by selenite and/or selenate resistant bacteria, which produce Se^0^ as a detoxifying method to survive in the presence of these salts [8,9]. Several metabolic pathways have been proposed to be involved in Se (IV) reduction by microorganisms, either in aerobic and/or anaerobic fashion. Se (IV) can be aerobically reduced to SeNPs by fumarate reductase and selenite reductase (SerT) [10]. Thiol-mediated Se reduction is the most widely recognized mechanism in which reduced thioredoxin can react with selenodiglutathione and form oxidized thioredoxin, reduced glutathione and selenopersulfide anion, from which Se^0^ is released [9]. However, other metabolic pathways include the thioredoxin reductase system [11] and siderophore-mediated reduction [12]. And even multiple mechanisms can be involved in Se (IV) reduction in a single bacterium strain depending on the concentration of Se to which they are exposed [13,14].

Most of the SeNPs produced by bacteria are spherical, with average sizes ranging from 10 to 700 nm [15] They are gaining popularity due to their low toxicity in humans, their high antioxidant, anticarcinogenic and antimicrobial capacity as well as their photoactivity [3,16]. Although SeNPs are known to have low specific surface area, they have shown to be good adsorbents due to their functional properties, that depends upon the surface hydrophobicity [11].

Biogenic SeNPs are always anchor to polymeric substances, such as proteins, polisaccharides and lipids [17], [18], [19]. These surface modifications make them stable and their suspensions well dispersed since they act as coating agents [14]. All these differences are attributed to the metabolic pathways involved in the SeNPs production by bacterial strains [20]. It is still unclear how the excretion of SeNPs takes place. There have been proposed the secretion in vesicles, and the cellular lysis as possible paths [10].

As has been mentioned before, SeNPs have high biocide activity. As recent examples, we can mention the observation made by Adibian et al. [21], these authors reported that SeNPs produced by a *Rosmarinus officinalis* extract had antimicrobial activity towards gram-positive and gram-negative bacteria. Furthermore, it has been established that SeNPs can prevent biofilm formation by pathogenic bacteria on medical supplies [4]. On the other hand, the antifungal activity of SeNPs has been investigated on the pathogenic fungal species, *Pyricularia grisea, Colletotrichum capsici* and *Alternaria solani,* of tomato, pepper and potatoes, respectively [22]. Moreover, it has been reported that SeNPs can inhibit the growth of *Aspergillus fumigatus* and *Candida albicans*, which produce human infections and are resistant to current antifungal agents [23,24]. However, to the best of our knowledge, the ability of biogenic SeNPs to inhibit the growth of wood-decay fungi has not yet been investigated.

*Nothofagus pumilio* (Lenga) is one of the predominant species in Patagonian forests, encompassing 1200,000 hectares of which 300,000 are productive forests for wood extraction [25]. Moreover, this species is the most relevant one regarding wood industry in South Patagonia of Argentina and Chile [26]. In south Patagonia, Lenga wood is often stored outdoors and in direct contact with the ground, thus being prone to fungal contamination. It has been reported that 85% of Lenga wood is rotten by fungi [27,28].

*Oligoporus pelliculosus* is a brown rot xylophagous fungi responsible for Lenga wood main economic losses. It is estimated that 80% of the fungal decay in wood building materials are due to this type of fungi [29]. New approaches for wood protection include, for example, the replacement of preservatives use by wood modification methods, to make this matrix no longer suitable as substrate for wood-decay fungi [30]. Other strategy suggested is to use antagonistic microorganisms to avoid these wood deceases. In this respect, Marfetán et al. [31] had reported that *Delftia* sp. 5 had a fungicide effect on *Phytophthora austrocedri*, a pathogenic oomycete which causes a root rot disease in *Austrocedrus chilensis,* another key species of Patagonian forests. The impregnation of wood with biocides is a novel strategy to improve wood durability. In this regard, wood impregnation with SeNPs could retard or even avoid sawn wood deterioration. In this work we aim to study the capacity of *Delftia* sp. 5 to produce SeNPs able to inhibit the growth of *O. pelliculosus* in Lenga sawn wood.

## 2 Materials and methods

### 2.1.1 Bacterial and fungus strains and growth media

*Delftia* sp. 5 was previously isolated from rhizosphere microorganism of *Austrocedrus chilensis* from Parque Nacional Los Alerces, Chubut, Argentina [31]. Briefly, *Delftia* sp. 5 was identified by using the 16S region primers 27f (5′-AGAGTTTGATCATGGCTCAG-3′) and 1492R (5′-TACGGTTACCTTGTTACGACTT-3′). Purification and sequencing of the PCR products were performed by the Macrogen Corp. The aligned matrices were deposited in TreeBASE under submission ID 25,799. *Delftia* sp. 5 nucleotide sequence data was analyzed using NCBI-BLAST (http://blast.ncbi.nlm.nih.gov/Blast.cgi) with the megablast algorithm. As a confirmation method a second analysis was made using ezBioCloud (http://eztaxon-e.ezbiocloud.net/). The genus of the assayed strain was also confirmed by microscopic analysis of fresh cultures and by Gram staining. The cultures were stored at −20 °C in Luria-Bertani (LB) with 20% (v/v) glycerol. Cultures were grown in LB at 30 °C with agitation (150 rpm) during 16 h prior to the experimental assay. On the other hand, the fungus *O. pelliculosus* 252 was isolated from rotten Lenga trees [27] and belongs to the *Herbario del Centro Forestal CIEFAP* (HCFP). The mycelial plugs of *O. pelliculosus* 252 were stored in sterile vials with sterile distilled water at 4 °C and grown in Potato Dextrose Agar (PDA) plates prior to experimental assay.

### 2.1.2 Microbial growth in the presence of selenite

Active cultures of the bacterial strain *Delftia* sp. 5 previously grown in LB were inoculated at 2% (v/v) in LB supplemented with filtered (0.22 µm membranes) and sterilized Na_2_SeO_3_ (Sigma-Aldrich Chemical Co., MO, USA) aqueous solutions at different concentrations (0, 10, 20, 40, 80, 160, 320, 640 mg *L*^−1^) in a microplate at 30 °C and 150 rpm during 48 h using a microplate reader (Multiskan Sky High, Thermo Scientific, Waltham, Massachusetts, USA). Differences in OD_600_ were recorded during 48 h to determine cell growth at different Se concentrations. The optimal Se concentration (as Na_2_SeO_3_) and incubation period were determined by following a previously reported method [1]. Briefly, since the development of reddish coloring is attributed to the formation of elemental selenium (principal component of SeNPs), the Na_2_SeO_3_ concentration that led to the maximum intensity of the reddish coloring was taken as the optimal one.

### 2.1.3 Synthesis of biogenic SeNPs by *Delftia* sp. 5

SeNPs synthesis and purification was carried out using the method reported by Medina Cruz et al. [32] with some modifications. Briefly, *Delftia* sp. 5 was grown in 50 mL of LB supplemented with 160 mg *L*^−1^ of Se (Na_2_SeO_3_) at 30 °C and 150 rpm during 48 h. Cells containing Se^0^ were isolated by centrifugation (5000 g, 20 min) washed with distilled water and cell pellets were mixed with 10 mL of Tris–HCl 0.1 M pH 6.7 and incubated at 90 °C during 1 h to release intracellular SeNPs by cell lysis. The SeNPs were isolated by centrifugation (14,000 g, 20 min), resuspended in the same volume of distilled water and filtered through 0.22 µm nylon membrane filters. Concentration of total selenium in the extracts was analyzed by ICP-MS after total digestion of the samples in closed vessels containing 1 mL of concentrated HNO_3_ and 0.5 mL of 30% (v/v) H_2_O_2_ using a microwave oven (MSP microwave oven, CEM, Matthews, NC, USA). The resulting solutions were cooled down, diluted to a 25 mL final volume with MilliQ water, and further analyzed for Se concentration with a Perkin Elmer NexION 350X ICP-MS with hydrogen gas as collision gas in the mass spectrometry facility of the *Centro de Estudios Fotosintéticos y Bioquímicos (CEFOBI)-CONICET,* Rosario, Argentina.

### 2.2 SeNPs characterization

#### 2.2.1 Detection of SeNPs in Delftia sp. 5 cultures by scanning electron microscopy (SEM)

The presence of SeNPs in the *Delftia* sp*.* 5 cell culture was confirmed by SEM with a SEM-FEG Nano Nova 230 at the *Departamento de Caracterización de Materiales, Centro Atómico Bariloche, Comisión Nacional de Energía Atómica* (S. C. de Bariloche, Argentina) microscopy facility. Fixation was done by suspending the cells with 2% (v/v) glutaraldehyde solution. Fixed cells were dehydrated through a series of alcohol dehydration steps (40, 60, 80 and 100%) and layered onto solid agar-coated SEM coverslips.

#### 2.2.2 Detection of SeNPs by transmission electron microscopy (TEM)

The suspensions obtained as indicated in section 2.1.3 were used to perform TEM determination of the SeNPs. A TEM FEI TECNAI F20 G2 placed at the *Laboratorio de Microscopía de Física de Materiales,* of the *Centro Atómico Bariloche, Comisión Nacional de Energía Atómica* (S. C. de Bariloche, Argentina) was used. Samples were prepared by placing a drop of each suspension onto a 300-mesh lacey carbon grid. The film on the TEM grids was allowed to dry for 5 min at room temperature. Analysis of SeNPs composition was carried out by EDS microanalysis. The average diameter of SeNPs was measured from the obtained images by using the free image-processing software, ImageJ (version 1.52a, Java 1.8.0_112, Wayne Rasband, National Institutes of Health, USA; website: https://imagej.nih.gov/ij/).

#### 2.2.3 Attenuated total reflectance Fourier transform infrared (ATR FT-IR) spectroscopic analysis of SeNPs

Infrared spectroscopic analysis was carried out at the Magnetic Resonance facilities of LiZys laboratory (S.C de Bariloche, Argentina) using a uART Spectrum two (Perker-Elmer FT-IR Spectrometer), in the spectral region from 4000 to 400 cm^−1^. The samples were dried with N_2_ flux, and the recorded spectra were analyzed using the Spectrum software (Perkin Elmer).

#### 2.2.4 UV–visible spectroscopic analysis of SeNPs

Absorption of SeNPs suspension was recorded in the UV-visible range at room temperature with a double beam PG Instruments Limited T90+ spectrophotometer, equipped with an integration sphere IS19–1 accessory. From this spectrum, the band gap of the material was determined by applying the Tauc's method for direct semiconductors. In addition, the spectrum of the supernatant after the centrifugation step to separate SeNPs from the cellular debris was recorded, as well as non-sonicated samples as a control (data not shown).

#### 2.2.5 Dynamic light scattering (DLS) and ζ-potential determination of SeNPs

Dynamic light scattering analysis was carried out at the Magnetic Resonance Laboratory facilities (LiZys, S.C de Bariloche, Argentina) using a zs90-Zetasizer (Malvern Instruments, Malvern, UK) equipped with a 633 nm helium–neon laser light source. The SeNPs samples were diluted with milliQ water to obtain a colorless solution, stabilized for 30 min, and analyzed at 25 °C. Each sample was measured three times using 70 channels in logarithmic scale with a size range between 0.4 and 1000 nm and an angle of 13° was used. The samples distributions were measured using the volume, intensity, and number parameters. The results were adjusted to the number distribution. The zeta potential measurements were done using the data obtained for the hydrodynamic diameter using an electrolytic cuvette and applying a 150 V potential.

#### 2.2.6 X-ray diffraction (XRD) profile of the SeNPs

X-ray diffraction (XRD) profile of the SeNPs was obtained in a diffractometer Bruker D8 Advanced, belonging to the *Departament de Fisicoquímica de Materiales – Centro Atómico Bariloche – CNEA (Argentina)*, with Cu-K_α_ radiation (0.15406 nm) in the ϴ−2ϴ geometry (from 10° to 90°) and at room temperature. For this, an aqueous solution containing the SeNPs in a relative high concentration was doped on a plan glass over a heater (60 °C). After the liquid evaporation, the powder was dispersed in a circle with 1.5 cm in diameter. The XRD data was analyzed with the software X`Pert HighScore.

### 2.3 Antifungal activity of SeNPs

#### 2.3.1 Antifungal activity of SeNPs in agar plates

The antifungal activity of the SeNPs was tested using a plate diffusion assay in PDA. Firstly, 5 mm mycelial plugs of *O. pelliculosus* 252, taken from the margin of a fifteen-days old culture, were placed in the center of 90 mm diameter Petri dishes and 50 mL of the following suspensions were placed in 5 mm wells, to determine their capacity for inhibit the fungus growth: a) SeNPs suspension, b) *Delftia* sp. 5 cell-free supernatants, and c) sodium selenite aqueous solution (34 mg L^-1^). A second assay was performed by spreading a 500 µL SeNPs suspension (or distillated water as a control) over PDA plates and, after drying, the *O. pelliculosus* 252 mycelial plugs were placed at the center of Petri dishes. In all the cases, the plates were incubated at 22 ± 2 °C for fifteen days during which the mycelial growth was measured at regular intervals.

#### 2.3.2 In vitro Lenga wood assay

Cross cut wood samples of Lenga of 2.0 × 2.0 × 0.5 cm were sterilized at 121 °C during 20 min in glass Petri dishes. Subsequently, wood samples were incubated with 15 mL of distilled water or SeNPs suspensions at room temperature for 16 h. After incubation, 5 mm mycelial plugs of *O. pelliculosus* sp. 252, taken from the edge of a fifteen-days old culture, were placed in the center of the wood samples. Samples were incubated at room temperature and humid atmosphere. Mycelial growth was monitored at regular intervals during 60 days by using a laboratory magnifying glass and an optical microscope.

### 2.4 Statistics

Studies were carried out in triplicate of independent assays and results were expressed as means with standard deviations. TEM and SEM images were done in duplicate and images were selected from independent assays. ICP-MS were performed for independent assays. Results were expressed as mean ± standard deviation (SD). Data were submitted to ANOVA general linear model, and pair-comparison of means was performed by Tukey's post hoc test. Student *t*-test was performed to compare paired samples. Statistical analysis was carried out by using Minitab® 17.1.0 software (Minitab, State College, PA, USA) and GraphPad Prism® 8.0 software (GraphPad Inc., San Diego CA, USA). The *P* < 0.05 was considered as statistically significant.

## 3 Results

### 3.1 Microbial growth in the presence of selenite and SeNPs synthesis by *Delftia* sp. 5

**Fig. 1:**
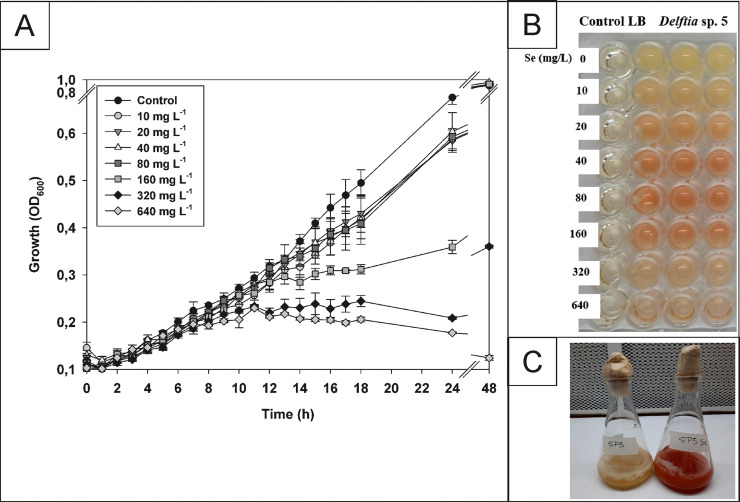
Growth of *Deltia* sp. 5 in LB (control) and in the presence of Se. A) Growth curve (absorbance at 600 nm) in LB supplemented with different concentrations of Se (0–640 mg *L*^−1^) at 30 °C during 48 h. B) Microplate showing the production of SeNPs (color change) in LB with Se (0–640 mg *L*^−1^) incubated at 30 °C during 48 h. C) Growth of *Delftia* sp.5 in LB (control) and LB with 160 mg *L*^−1^ of Se at 30 °C and 48 h incubation.

Similar OD_600_ values were observed when *Delftia* sp. 5 was grown in LB with selenite concentrations between 0 and 160 mg *L*^−1^ after 48 h incubation. However, when the strain was grown with 160 mg *L*^−1^ of selenite, the growth rate decreased 69% between 12 and 24 h incubation comparing to control in LB. Moreover, when 320 mg *L*^−1^ of selenite was added, the OD_600_ after 48 h was 3-fold lower than the observed when selenite concentration was below 160 mg *L*^−1^ (Fig. 1A). On the other hand, the maximum reddish color (indicating SeNPs formation) was observed in the microplate when using a selenite concentration of 160 mg *L*^−1^ (Fig. 1B), which was also observed in the 50 mL Erlenmeyer's cultures after 48 h incubation (Fig. 1C). In addition, the growth media remained yellow when the strain grew in the absence of selenite and no growth or color changes were observed in the non-inoculated LB either with or without selenite addition.

*Delftia* sp. 5 cells biotransformed 6.290 ± 0.163 mg *L*^−1^ of the Se added (as Na_2_SeO_3_) into insoluble Se^0^ when grown in LB with 160 mg *L*^−1^ at 30 °C during 48 h. The Se concentration of SeNPs concentrated and recovered after cell lysis and filtration (through 0.22 µm filter) was 33.60 ± 0.107 mg *L*^−1^ (mainly as red Se^0^) as detected by ICP-MS.

### 3.2 SeNPs physicochemical characterization

**Fig. 2:**
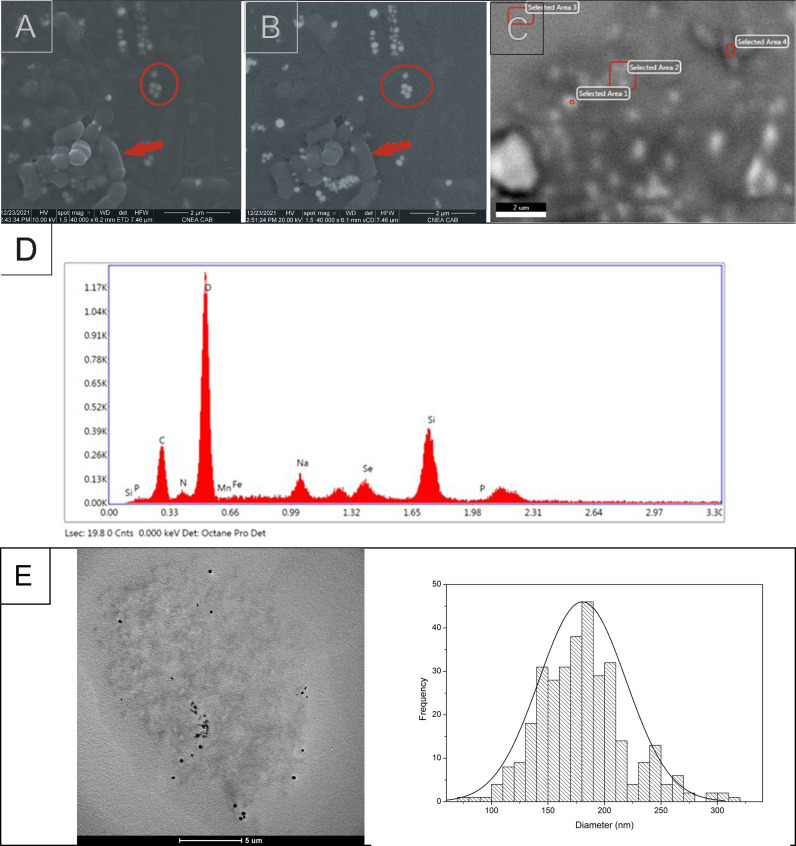
SEM microphotographs of *Delftia* sp. 5 grown in LB with 160 mg *L*^−1^ of Se incubated at 30 °C and 150 rpm during 48 h. A) Imagines using secondary electrons. B) Images obtained by backscattered electron reflectivity. In both imagines (A and B) SeNPs are marked with circles and *Delftia* sp. 5 cells by arrows. C) Selected area for Energy Dispersive Spectroscopy (EDS). D) EDS analysis graph depicting energy on X-axis and number of counts on Y-axis representing elemental composition derived from the selected area. E) TEM images and PSA histogram of SeNPs.

SEM images showed the presence of spherical SeNPs in the *Delftia* sp. 5 culture by using secondary electron detection (Fig. 2A) and also by backscattered electrons detection (Fig. 2B). Although other elements were also detected by SEM (Fig. 2C, D), their reflective power was lower than for Se indicating that the sparkly particles observed in Fig. 2B corresponded to Se-containing NPs. The presence of Se was also confirmed by the detection of the L signal of Se by EDS, other elements such as carbon, nitrogen and oxygen were also found in the nanoparticles, while the Si signal corresponded to the material of the detector used (Fig. 2D). On the other hand, the presence of SeNPs in the cell free extracts was observed by TEM and the corresponding EDS microanalysis confirmed the presence of Se [Lα (1.4 keV), Kα (11.22 keV), and Kβ (12.49 keV)] in the nanoparticles. The average diameter size of the SeNPs detected was 180.5 nm (Fig. 2E).

**Fig. 3:**
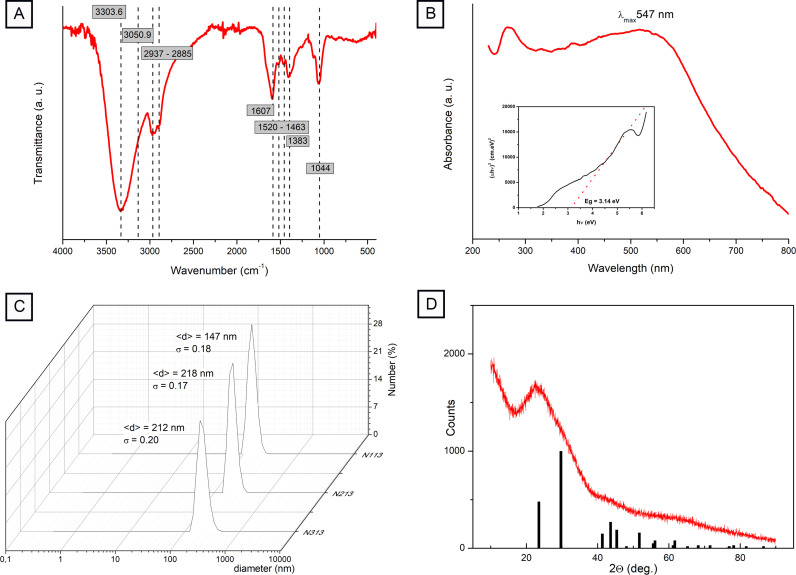
SeNPs characterization: A) Attenuated Total Reflectance Fourier Transform Infrared (ATR FT-IR). B) UV-visible spectroscopic analysis of SeNPs. C) Dynamic light scattering (DLS). D) X-ray diffraction (XRD) profile.

The results obtained by ATR FT-IR spectroscopy were consistent with SEM/EDS results since C, O and N were detected, and showed information about the functional groups present on SeNPs surface (Fig. 3A). Wavenumbers of the maxima for the main bands in the ATR FT-IR spectra were summarized in Table 1 together with their tentative assignment [18,33,34].

**Table 1:** Wavenumber of the main bands in the ATR FT-IR spectrum of SeNPs isolated from *Delftia* sp. *5* cultures (see Fig. 3A) and their tentative assignment.

O-H, ν^a^; N-H (Amide A in proteins), ν ^b^	C—H in -CH_2_, ν_as_	C-H in -CH_2_, ν_s_	C=O and C—N (Amide I (in proteins), ν^c^	N-H and C—N (Amine II in peptides and proteins), ν	—CH_3_, δ (in proteins, lipids, polyesters, etc.)	-COO^−^, ν_s_	C = O, ν in aminoacids
3303 3050 (sh)	2936	2885	1607	1520	1463	1380	1045

Designations: ν – stretching vibrations; ν_s_ – symmetric stretching vibrations; ν_as_ – antisymmetric stretching vibrations; δ – bending vibrations; sh – shoulder.

^a^ from water molecules, alcohols and/or phenols [34].

^b^ and also from peptides and/or polysaccharides [33].

^c^ and also from N—H linkages of amines [18].

UV-visible spectrum of isolated SeNPs showed a broad plasmon band centered at ca. 600 nm which is indicative of an average particle size of 180 nm and consistent with the result obtained from the analysis of electronic micrographs. Moreover, a less intense band centered at 260 nm is observed, due to absorption of DNA conjugated double bonds anchored to SeNPs surface. From the Tauc's representation of the data, treating Se as a direct band gap semiconductor, a value of E_g_ = 3.139 eV was determined (Fig. 3B). Whereas by means of TEM images as well as the position of the plasmon band in the UV-visible spectrum of the SeNPs suspension an average diameter of 180 nm was obtained, when analyzing the DLS results, an average hydrodynamic diameter of 192.33 ± 8.6 nm was observed. The measured ζ-potential was – 41.4 ± 1.3 mV (Fig. 3C).

The diffractometer of the SeNPs clearly shows the presence of broad baseline, which should be associated to the presence of a background from the glass substrate and the organic material present in the sample (Fig. 3D). In addition, a broad peak between 2ϴ = 23° and 35° was clearly observed. The vertical lines indicate the position and relative intensity of the diffraction peaks expected for the Se (reference code 01–086–2246 - Hexagonal, Space group: P3121, *a* = 0.43680 nm, *b* = 0.43680 nm and *c* = 0.49580 nm). As observed, the main peaks of the Se localized at 23.499° and 29.681°, with relative intensity of 0.48 and 1, respectively, coincides with the broad peak observed in the experimental data. Consequently, it can be indexed to the peaks (100) and (101), and its broad nature is probably related to the small size of the crystallite in the nanoparticles, as described by the Scherrer equation: *L* = Kλ/β.cosθ, where L is the crystallite size, K is a shape factor; λ is the incident wavelength and β the peak width. Then, XRD results evidence the Se as the exclusive crystalline phase in the sample as well as the reduced dimension of the crystallite.

### 3.3 Antifungal activity of SeNP and in vitro *Nothofagus pumilio* (Lenga) wood assays

**Fig. 4:**
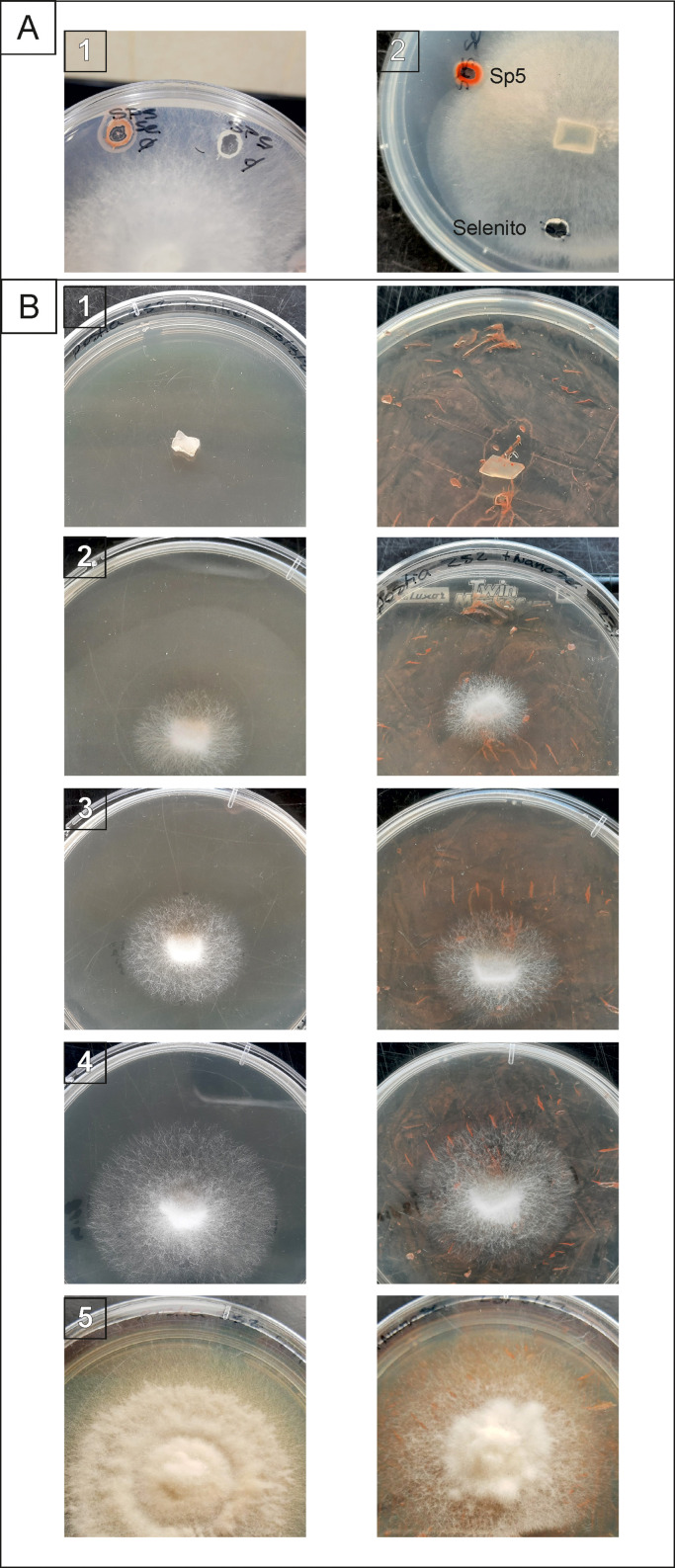
In vitro antifungal activity of biogenic SeNPs produced by *Delftia* sp. 5 against *O. pelliculosus* 252 on PDA plates. A). Plates showing fungal growth inhibition using wells by 1). SeNPs produced by *Delftia* sp. 5 (SP5 Se) and the cell free supernatant (SP 5) and 2). SeNPs produced by *Delftia* sp. 5 (SP5 Se) and sodium selenite (Se). B). Plates showing *O. pelliculosus* 252 inhibitions when SeNPs were spread over the agar. 1., 2., 3., 4 and 5 correspond to incubation times 0, 5, 6, 8 and 13 days. Control samples (left) and SeNPs treated (right).

Fungal growth inhibition was observed when the SeNPs were added in the PDA wells (Fig. 4A), while no inhibition was detected when the controls cell-free supernatants of *Delftia* sp. 5 and sodium selenite were used. On the other hand, when SeNPs were spread over the agar a negative effect on the fungal growth was observed (Table 2 and Fig. 4B). The highest inhibition effect was achieved after 13 days in the samples treated with SeNPs suspensions. The *O. pelliculosus* 252 hyphae were 15 mm smaller and noticeably less dense in the SeNPs treated plates respect to the control ones.

**Table 2:** Percent inhibition of *O. pelliculosus* at different times of mycogenic SeNPs produced by *Delftia* sp. 5.

Time (days)	Mycelial diameter (mm)		% of inhibition
	Control	SeNPs	
0	5.0 ± 1.0	5.0 ± 2.0	–
5	15.0 ± 1.0 ^a^	12.0 ± 2.0 ^a^	16.25 ± 5.30
6	35.0 ± 3.0 ^b^	25.0 ± 1.0 ^c^	32.71 ± 5.85
8	53.0 ± 1.0 ^d^	44.0 ± 5.0 ^d^	11.37 ± 7.93
13	85.0 ± 4.0^e^	60.0 ± 2.0 ^f^	28.90 ± 0.72

Values are the means of three replicates ± SD, means followed by the same letter(s) within the row are not significantly different according to Tukey's Honestly Significant Difference.

**Fig. 5:**
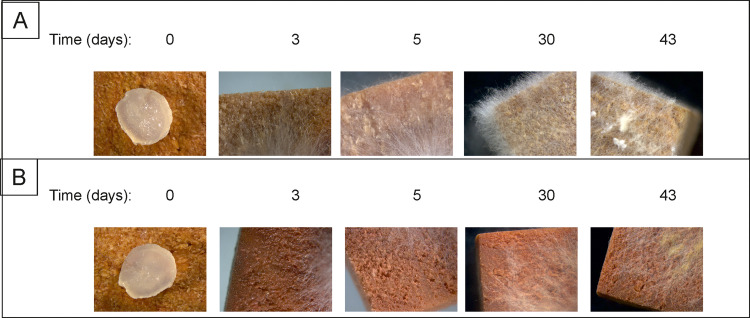
Antifungal activity of biogenic SeNPs produced by *Delftia* sp. 5 against *O. pelliculosus* 252 on A) control lenga pieces (embedded in sterilized water) and B) lenga pieces embedded in SeNPs solution.

**Fig. 6:**
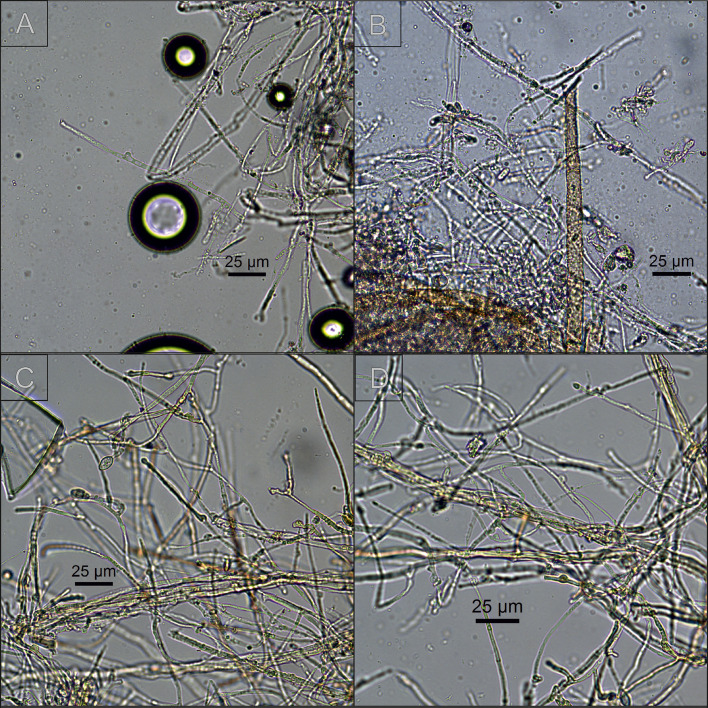
Microscopic observation of *O. pelliculosus* incubated on Lenga wood pieces during 43 days. A and B) wood pieces previously embedded in water (control). C and D) wood pieces previously embedded in SeNPs solution.

Encouraged by these results, SeNPs suspensions were used to impregnate Lenga wood samples (Fig 5). After 3 days of incubation, *O. pelliculosus* 252 hyphae in the control wood samples (incubated with water) managed to reach the edges of the wood pieces; whereas for the SeNPs treated samples, the fungal growth was retarded, and hyphae reached the edges only after a 30-days incubation period. In addition, after 43 days the fungus completely colonized the control wood samples, whereas in the SeNPs treated samples the growth of *O. pelliculosus* 252 was retarded. Finally, microscopic images of control wood samples showed healthy hyphae with chlamydospores and fibulae, while in SeNPs treated wood samples *O. pelliculosus* 252 growth was retarded (Fig. 6).

## 4 Discussion

SeNPs are usually produced by reduction of selenite using chemical or enzymatic methods [1]. However, the SeNPs produced by chemical methods generate toxic by-products and the yield nanoparticles can suffer photocorrosion [14]. An alternative to chemical synthesis, is the production of biologically synthesized SeNPs by bacteria which are resistant to oxyanions and reduce selenium salts to elemental Se [15]. This process can be enzymatic or not and SeNPs can remain inside the periplasm or be excreted [35]. Selenium reducing bacteria have been isolated from diverse environments and in general phylogenetically diverse microorganisms isolated from pristine and/or contaminated areas had shown the highest efficiency for reducing oxyanions [36]. In this respect, Wadgaonkar et al. [37] isolated a *Delftia lacustris* strain from a casual contamination of a 7.896 g *L*^−1^ selenate stock solution which resisted a Se concentration (as selenite) of 1970 mg *L*^−1^. However, Bautista-Hernandez et al. [38] reported that a *Delftia tsuruhatensis* isolated from a mine tailing in El Oro de Hidalgo and Zacualpan (Mexico), was able to resist a Se concentration (as selenite) of 237 mg *L*^−1^. In this work we observed that *Delftia* sp. 5 (isolated from Parque Nacional Los Alerces, Chubut, Argentina) could grow poorly when Se concentration was above 320 mg *L*^−1^ and that the higher SeNPs production without dramatically affecting the cell growth was achieved at a Se concentration of 160 mg *L*^−1^ (as selenite). *Delftia* sp. 5 was able to biotransform 3.9% of the 73.0 mg *L*^−1^ added selenite (160 mg *L*^−1^ Se) into insoluble selenium while for *D. lacustris* an 8.6% of insoluble selenium was found in the cell pellets when the cells were incubated with 86.0 mg *L*^−1^ of selenite. The differences on the Se biotransformation into insoluble Se found between *Delftia* sp. 5 and *D. lacustris* could be due in part to addition of lactate in the culture media used by Wadgaonkar et al. [37] in contrast to the LB medium used in this work, since it has been reported that different carbon sources (lactate, mannitol, propionate) can be used as electron donor for selenite reduction by bacteria cells [39]. However, the biochemical mechanisms for aerobic selenite reduction to Se^0^ is still not completely elucidated and it has been attributed to different enzymes (membrane associated reductases, thioredoxin reductase system, siderophore mediated reduction, etc.;). *Delftia* sp. 5 produced spheric Se nanoparticles as a consequence of selenite reduction and no detrimental effects were observed on the cell structure (SEM images) although, a lower growth rate was observed when 160 mg *L*^−1^ of Se were added. Similar results were observed for other SeNPs producing bacteria [9].

The presence of an organic layer in the SeNPs produced by *Delftia* sp. 5 was confirmed by the ATR FT-IR analysis which showed features that could be attributed to proteins and carbohydrates. In this respect, the peaks in 2936 and 2885 cm^−1^ could correspond to -CH_2_ moieties from peptides and proteins, since when lipids are present, there is a trend to observe a band ca. 1200 cm^−1^ due to PO_2_^−^ groups from phospholipids [40]. In addition, another lipid related band in 1725 cm^−1^ (due to C=O stretching in ester groups) was absent in the spectrum of SeNPs produced by *Delftia* sp. *5*. The bands located in 1463 cm^−1^ and 1045 cm^−1^ was assigned to -CH_3_ groups from protein alkyl residues and to C—O stretching vibration of amino acids instead of to C—O-C stretching in carboxylic acids and ester groups [[18], [33], [41]]. Consistently, Kamnev et al. [42] have reported that in nutrient stressing conditions, *A. brasilense* Sp7 tend to accumulate reserve biopolyesters; but no signal of the production of these biomolecules was observed when the bacteria were incubated in the presence of 10 mM Na_2_SeO_3_, indicating that the bacterial metabolism switched to reduce selenium (IV) to Se^0^ (with the formation of SeNPs) without noticeable lipids biosynthesis. Furthermore, the broad band in 1380 cm^−1^ assigned to the symmetrical stretching of the C=O bond from carboxylic groups (-COO^−^) could explain the negative surface potential observed for these SeNPs, in agreement with results observed in other biogenic SeNPs previously reported [10]. SeNPs showed a semiconductor value of E_g_ = 3.139 eV. Values reported in the literature range from 2.7 eV for bulk Se and amorphous Se films [42], 2.75 eV for SeNPs between 45 and 90 nm [43], 2.7 – 3.1 eV for crystalline SeNPs [44], and from 3.28 to 3.78 eV for SeNPs between 10 and 18 nm [[45], [46]]. On the other hand, the ζ-potential was – 41.4 ± 1.3 mV observed agrees with various biogenic SeNPs produced by several microorganisms and can be due to the -COO^−^ groups exposed in the SeNPs surface [5,14]. In this respect, Piacenza et al. [14] reported that biogenic SeNPs were more efficient in inhibiting pathogenic bacteria and yeast than synthetic SeNPs due to the surface component of the former NPs. Furthermore, Filipović et al. [47] demonstrated that the surface chemistry was the most influenced parameter that determines the antimicrobial activity of SeNPs. Moreover, these authors showed that the most stable SeNPs were those with highly negative zeta potential (SeNPs-gluc with −45 mV). The antifungal activity of biogenic and chemically synthesized SeNPs has been reported by different authors [22,23]. Moreover, Bafghi et al. [24] reported that SeNPs synthesized using plant extracts could inhibit the growth of drug resistant *Aspergillus* and *Candida* species. SeNPs produced by *Delftia* sp. 5 were able to reduce the hyphae density of the brown rot fungus *O. pelliculosus* 252 grown on PDA after 13 days incubation. In this respect, Wildermuth and Rovira [48] stated that the hyphae density was positively related to the severity of the wood disease.

Brown rot depolymerize cellulose rapidly at the beginning of wood colonization causing strength losses even at early stages. Indeed, brown rot inhibition is a greater challenge than white rot for wood in service outdoors [30]. Biogenic SeNPs are a good alternative for surface protection because of their high biocidal effect with a minimal toxicological effect comparing to other NPs such as e.g.*,* AgNPs [24]. Moreover, impregnation of biocides within the wood is nowadays the most used method 2015; [30]. In this respect, the use of SeNPs as biocide is an excellent strategy for preventing wood rotten, since it is known that this material has low toxicity, low pollutant load, is chemically stable and its production is inexpensive [49]. In the Lenga assay wood pieces were embedded with the SeNPs suspension and the wood color changes to red indicating that the SeNPs were probably on the surface of the wood. The radial growth of *O. pelliculosus* 252 in the wood was retarded in the SeNPs embedded samples. Similarly, De Filpo et al. [50] observed that wood impregnation with photoactive titanium NPs could prevent the growth of two fungi responsible for fast wood decay when irradiated under UVA light. They report a band gap of 3.2 eV for the titanium NPs used, which is in the order of the value reported here for SeNPs (3.139 eV). Even further, they report the impregnation of the nanoparticles in the presence of a surfactant to assist on particle dispersion within the wood samples. Both incorporations, of irradiation and surfactant use, are still to be prove for our system.

The mechanism by which SeNPs inhibit fungal growth is still unknown, however Se sulfide has been used to treat fungal infections in humans [23]. It has been stated that Se can act over DNA and bacterial protein synthesis [51]. Furthermore, there is evidence that inorganic Se and organic diselenides and methyl-selenide can disrupt pathogen membranes by generating free radicals when reacting with thiol groups of membrane proteins [52]. However, none by EDS microanalysis when coupled with TEM nor SEM instruments showed any evidence of the presence of sulfur nor metal elements. And these elements were neither suggested by ATR FT-IR spectroscopy results. This absence is noteworthy since there is evidence that, among the group of bacteria able to reduce selenite into Se^0^ under aerobic conditions, exists organisms that biotransform it by producing Se particles containing sulfur [53], as well as metal selenides and methylated selenium species [54].

Although in the present work we show the ability of SeNPs to inhibit the main brown rotting fungi *O. pellicullosus*, the mechanism by which these SeNPs inhibit fungal growth must be elucidated in future works. Moreover, it would be interesting to evaluate the capacity of these SeNPs to inhibit the growth of other wood-decay fungi of economically important tree species for the wood industry, as well as to study the chemico-physical properties of the wood after SeNPs impregnation to ascertain this treatment as a protection treatment and its need of maintenance.

## 5 Conclusions

*Delftia* sp. 5 produced spherical Se^0^ nanoparticles by selenite reduction. These SeNPs showed an average size of 180 nm as measured by DLS and micrographic TEM and SEM images. The presence of an organic layer in the SeNPs produced by *Delftia* sp. 5 was confirmed by the FT-IR analysis which showed features that could be attributed mainly to proteins and carbohydrates. The impregnation of the sawn wood with the SeNPs produced by *Delftia* sp. 5 before drying could prevent wood decay by inhibiting the growth of deteriorating fungi such as *O. pelliculosus*, one of the main fungi found in Lenga rotten trees [27]. These are promising results that set the fundamentals to continue studying the metabolic routes involved in the bacterial reduction of selenite to Se^0^ (as well as the mechanisms by which SeNPs inhibit fungal growth), and all the aspects related to wood nanoengineering from a physico-chemical point of view.

## Disclosure statement

No conflicts of interest.

## Author contributions

All authors read and take responsibility for the integrity of the work as a whole and give their approval for this version to be published

## Data availability

No data was used for the research described in the article.

## Funding

This work was supported by projects PICT-2019-03437 of FONCyT and Strategic Program CIEFAP P7 A2 005 from CIEFAP, Argentina

## CRediT authorship contribution statement

**Micaela Pescuma:** Conceptualization, Methodology, Validation, Formal analysis, Investigation, Visualization, Writing – review & editing, Supervision. **Francisca Aparicio:** Conceptualization, Methodology, Resources, Software, Visualization, Investigation, Writing – review & editing. **Roberto D. Zysler:** Methodology, Resources, Data curation, Formal analysis. **Enio Lima:** Methodology, Resources, Data curation, Formal analysis. **Claudia Zapata:** Methodology, Resources, Data curation, Project administration, Funding acquisition. **Jorge A. Marfetán:** Formal analysis, Investigation, Writing – original draft. **M.Laura Vélez:** Formal analysis, Investigation, Writing – original draft. **Omar F. Ordoñez:** Conceptualization, Methodology, Resources, Writing – review & editing, Supervision, Project administration, Funding acquisition.

## Declaration of Competing Interest

Authors have no conflicts of interest to disclose.

## Data Availability

Data will be made available on request.
